# The Prevalence of Fear of Childbirth and Its Association With Intolerance of Uncertainty and Coping Styles Among Pregnant Chinese Women During the COVID-19 Pandemic

**DOI:** 10.3389/fpsyt.2022.935760

**Published:** 2022-06-27

**Authors:** Lingli Han, Hua Bai, Bing Lun, Yanxia Li, Yingfan Wang, Qingnan Ni

**Affiliations:** The Third Affiliated Hospital of Zhengzhou University, Zhengzhou, China

**Keywords:** childbirth fear, intolerance of uncertainty, coping styles, COVID-19, pregnant women

## Abstract

**Background:**

Fear of childbirth (FOC) is one of the most common psychological symptoms among pregnant women and significantly relates to cesarean section, anxiety, and depression. However, it is not clear the prevalence and risk factors of FOC among Chinese pregnant women since the outbreak of the COVID-19 pandemic.

**Aims:**

The objective of this study was to examine the associations between coping styles, intolerance of uncertainty, and FOC.

**Method:**

From December 2021 to April 2022, a cross-sectional survey was conducted in two hospitals in China through convenient sampling. The cross-sectional survey was conducted among 969 pregnant women, which included the Childbirth Attitude Questionnaire (CAQ), Intolerance of Uncertainty Scale-12 (IUS-12), and Simplified Coping Style Questionnaire (SCSQ).

**Results:**

The total prevalence of FOC was 67.8%. The percentages of women with mild (a score of 28–39), moderate (40–51), and severe FOC (52–64) were 43.6, 20.2, and 4.0%, respectively. The regression results indicated that primiparas, unplanned pregnancy, few spousal support, intolerance of uncertainty, and negative coping styles were significant risk factors of FOC. Women who adopt positive coping strategies experienced a lower level of childbirth fear.

**Conclusion:**

These findings suggest that cultivating positive coping styles and obtaining sufficient childbirth information may be helpful for mothers' mental health. Regular screening assessment of perinatal psychological symptoms, such as the high level of intolerance of uncertainty and negative coping styles, should be adopted to reduce the risk of fear of childbirth.

## Introduction

Fear of childbirth (FOC), a spectrum of anxious thoughts and feelings relating to woman's appraisal of labor and birth, was reported to be a prevalence of 14% around the world (1–4). Prior research has found that mild or high levels of FOC are correlated with prolonged labor, cesarean section, choice of epidural analgesia, prenatal and postpartum depression, and anxiety (5–9). The potential risk factors of FOC (e.g., young age, low educational level, anxiety, depression) have been identified in different countries or regions (8, 10–12). Additionally, Rondung et al. suggested that intolerance of uncertainty (IU) was one of the best predictors of FOC (13). However, the relationships among FOC, IU, and other variables are seldom investigated clearly in China during the COVID-19 pandemic.

Emerging evidence has shown that the COVID-19 outbreak had a significant psychological impact on pregnant women (14–16). Temporary closure of public places, strict quarantine policies, and other measures were taken in various countries to prevent the spread of the virus (14, 17–19). There was a lot of available negative news information about COVID-19 online, which may increase distress and fears for the users. A prior study revealed that users interested in suicide-related news are more likely to search it through various applications (20). Furthermore, it is confirmed that pregnant women have experienced more psychological symptoms since the outbreak (21). In addition, the extreme uncertainty caused by the COVID-19 pandemic elevated anxiety and fears among pregnant women (21–24). Baldessarini et al. also suggested that affective-temperament ratings, which were related to psychological distress and negative clinical outcomes, were higher in females (25). Thus, pregnant women may experience significant distress as they have continuously faced ambiguous circumstances during the pandemic.

Intolerance of uncertainty (IU) is defined as the tendency to react negatively to uncertainty, and it is a potential important transdiagnostic factor related to multiple psychological disorders in fear of childbirth (26–30). Previous studies have investigated that IU has robust associations with a range of disorder symptoms, including generalized anxiety disorder, social anxiety disorder, panic disorder, and agoraphobia (26, 27, 31). People with high levels of intolerance of uncertainty tend to experience greater physiological distress and avoidance of uncertainty (23, 32–36).

In addition, COVID-19 is an exemplar of a real-world uncertain and threatening situation related to uncertainty distress (37). Moreover, anxiety sensitivity significantly increased individuals' COVID-19 worries and behaviors, especially those with high IU (38). Emerging evidence suggests that individuals with high IU may take different threat reactivity strategies (e.g., internet searches and avoidance) to adjust to changing information about COVID-19 threats (39, 40). Meanwhile, pregnant women with high IU usually adopt avoidance strategies to cope with stress, which failed to improve the current situation (16, 26, 27). Consequently, an appropriate coping response (positive coping styles) toward uncertainty may protect pregnant women from the potentially detrimental impacts of the COVID-19 pandemic.

In Lazarus and Folkman's transactional coping theory (41), coping was defined as continuously altering cognitive and behavioral efforts to respond to stressors. The coping style included two widely known primary functions: emotion-focused coping (e.g., passive or active avoidance, escaping and positively reappraising the stressor) and problem-focused coping (e.g., seeking practical or informational support). It is of great significance to explore the relationships between psychological risk factors and coping styles of pregnant women during the pandemic to help them deal with stressors effectively. However, to the best of our knowledge, studies elucidating the relationships of IU, coping styles, and FOC in Chinese pregnant women are limited.

Given that intolerance of uncertainty and different coping styles have been well documented as predictors for individuals' mental health, it would be of great value to investigate the relationships among IU, FOC, and coping styles of pregnant women during the COVID-19 pandemic. In addition, the examination of risk factors related to FOC is essential for researchers to develop efficient interventions to improve pregnant women's mental health and ameliorate the erosion of the distress caused by the pandemic.

Therefore, the underlying hypothesis was that intolerance of uncertainty and negative coping styles would be positively related to fear of childbirth, but positive coping styles would be inversely correlated with IU and FOC in Chinese pregnant women. This study aims to investigate the prevalence and risk factors of FOC in Chinese pregnant women during the COVID-19 pandemic.

## Materials and Methods

### Design

From December 2021 to April 2022, a cross-sectional survey was conducted in two hospitals in China through convenient sampling. All participants were informed of the study's purpose and required to provide an informed consent form online before enrollment. The online platform of Wenjuanxing (https://www.wjx.cn/app/survey.aspx) was employed to distribute the electric questionnaires, which indicated to the participants when they had unanswered questions.

This survey included the sociodemographic characteristics questionnaire, the Childbirth Attitude Questionnaire (CAQ), the Intolerance of Uncertainty Scale-12 (IUS-12), and the Simplified Coping Style Questionnaire (SCSQ). Finally, there were 969 eligible samples in the data analyses.

### Study Participants

Inclusion criteria comprised being over 20 years of age, pregnant at 12–40 weeks with no severe gestational complications, no history of severe physical illness, fluent in Mandarin, and access to a smartphone.

Women with diagnoses of threatened abortion and fetal anomaly were excluded. All pregnant women need to provide online written informed consent at the beginning of this survey.

### Measurements

#### Sociodemographic and Obstetrical Characteristics Questionnaire

According to previous reviews and research (2, 10, 11, 42–44), we constructed the sociodemographic and obstetrical characteristics questionnaire to collect general information of pregnant women. It included age, employment status, educational level, monthly income (CNY), marital status, residence, medical insurance, gestational week, parity, planning of pregnancy, family's opinion of a childbirth mode, the preferred mode of childbirth, prenatal spousal support, access to childbirth information, and a regular prenatal visit. And the mode of previous birth and epidural analgesia during the last labor were added to it for the data collection of multiparous women.

#### Childbirth Attitude Questionnaire

The Chinese version of the Childbirth Attitude Questionnaire (CAQ) (45) was employed in this study. It was first designed by Areskog (46) and developed by Lowe (47) and Tanglakmankhong (48). Then, Wei et al. (45) translated it into Chinese and found four dimensions of the CAQ: fear of baby safety (FBS), fear of labor pain (FLP), fear of losing control (FLC), and fear of environment (FE). The CAQ consisted of 16 items scored on a 4-point Likert scale (1 “never” to 4 “high”). Scores of 16–27, 28–39, 40–51, and 52–64 represent mild, moderate, and severe fear of childbirth. It was reported good internal consistency reliability (Cronbach's α = 0.910) (45). Internal consistency of CAQ in this survey also showed excellent (Cronbach's α = 0.942).

#### Intolerance of Uncertainty Scale−12

Freeston et al. (29) first proposed the term intolerance of uncertainty and constructed the Intolerance of Uncertainty Scale (IUS) in 1994. Then, Carleton et al. (49) simplified it into the IUS-12 in 2007. The IUS-12 scored on a 5-point Likert scale (1, “not at all characteristic of me” to 5, “entirely characteristic of me”). Wu et al. (50) translated the IUS-12 into Chinese and investigated the IUS-12 consists of three dimensions: prospective action (PA), inhibitory action (IA), and prospective emotion (PE). In the Chinese IUS-12, Cronbach's alpha reliability coefficient was reported to be 0.79 in 1,275 Chinese adolescents. The Cronbach's α of IUS-12 was 0.853 in this study.

#### Simplified Coping Style Questionnaire

Xie compiled the SCSQ in 1998 (51), and it was widely used to assess coping styles in China. Xie simplified the ways of coping questionnaire (WCQ), first designed by Folkman and Lazarus (41). The SCSQ is a universal self-rating scale with 20 items and includes two subscales: positive coping (SCSQ-P) and negative coping (SCSQ-N). It scored on a 4-point Likert scale (0, “do not use” to 3, “often use”). The higher the sum score of the positive coping dimension was, the more pronounced the participants' propensity to adopt it and *vice versa*. The SCSQ showed good internal consistency (Cronbach's α = 0.868) in this study, as well as its sub-scales: 0.900 (SCSQ-P) and 0.837 (SCSQ-N).

### Data Collection

Trained midwives conducted the prenatal survey at the Perinatal Health Care Clinics of two tertiary hospitals in Henan Province, China. The online platform Wenjuanxing was employed to complete the survey. It will automatically generate a QR code or URL link for the manually entered questionnaire.

**Figure 1:**
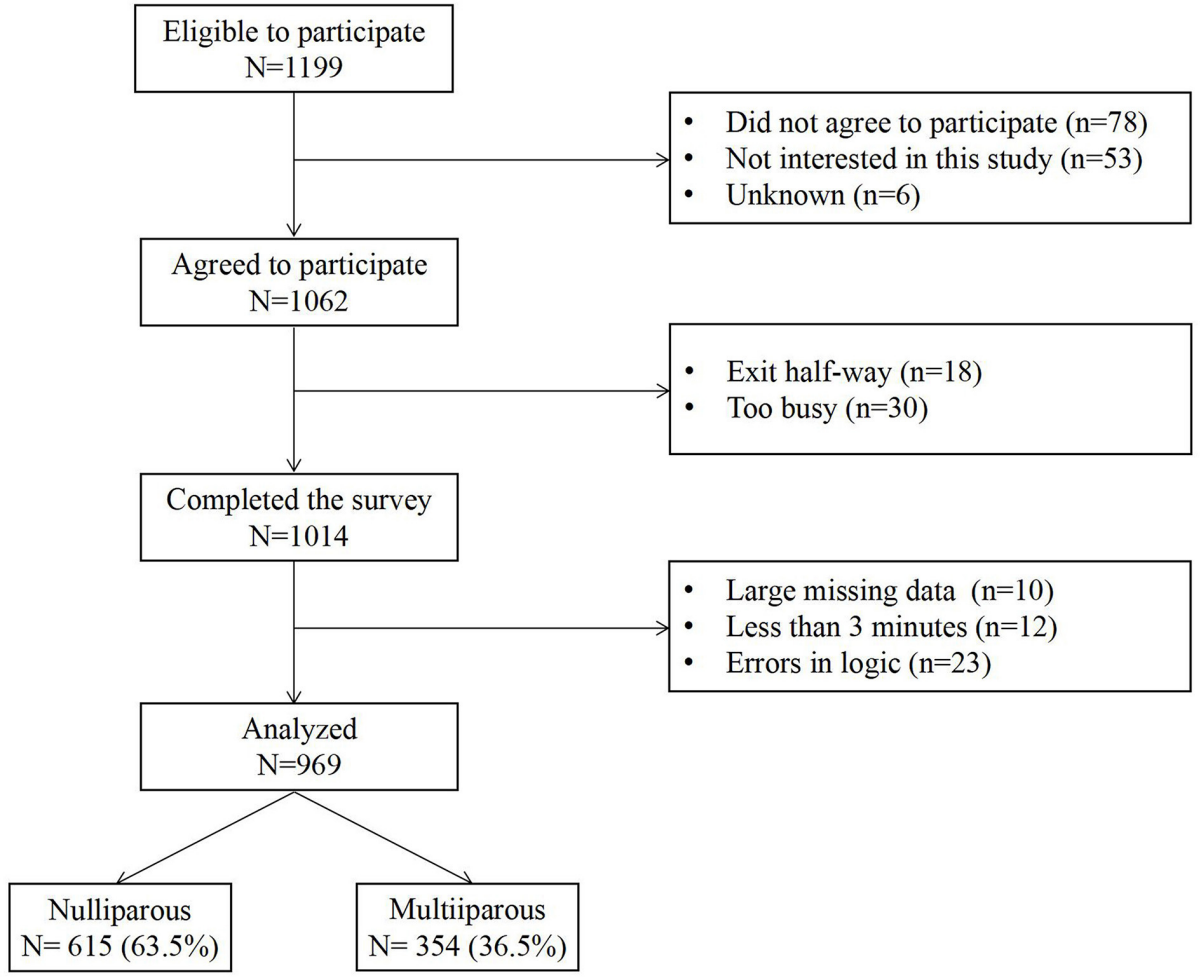
A flow chart of the participants.

During the survey, the midwives showed pregnant women the printed picture of the questionnaires' QR code or sent the URL link using WeChat. Then the participants scanned the QR code or clicked on the link to fill in the questionnaire within 15 min. There were no missing data because the survey was conducted through Wenjuanxing, which indicates to the participants when they had unanswered questions. Furthermore, all the collected data were manually checked for the questionnaire answer time (<3 min was excluded) and whether the answer was logical and reasonable (e.g., answering regularly or choosing the same option was excluded). The flow chart of the participants is shown in Figure 1.

### Data Analysis

Data were analyzed using the Statistical Package for Social Sciences (SPSS, version 25.0 for Windows). The participants' sociodemographic characteristics and the scores of CAQ, IUS-12, and SCSQ were presented with the frequencies, percentages, means (M), standard deviations (SD), and range of scores. Chi-square tests were used to explore the group differences in CAQ scores (CAQ < 28 and CAQ ≥ 28), as appropriate. Correlation analysis was performed to examine the relationships between FOC and independent variables, including demographic characteristics and the sum scores of CAQ, IUS-12, and SCSQ.

According to previous reviews and research (2, 10, 11, 42–44), age, parity, gestational weeks, and spousal support were associated with FOC. Thus, the exploratory analysis was first conducted to clarify the relationships between sociodemographic and obstetric factors and FOC. After these preliminary analyses, the multiple hierarchical linear regression for predicting FOC was conducted to investigate whether intolerance of uncertainty and coping style could significantly affect FOC. With the scores of SCSQ-P, SCSQ-N, and IUS-12 as independent variables and the score of CAQ as the dependent variable, the hierarchical linear regression analysis for predicting FOC was constructed by enter method.

The multiple linear regression analysis was established in three steps using the score of CAQ as the dependent variable, and the independent variables were entered in the following steps: (1) sociodemographic and obstetric factors (e.g., age, educational level, and parity); (2) the score of SCSQ-P; (3) the score of SCSQ-N; (4) the score of IUS-12.

In addition, we compared the mean differences across the scores of CAQ, SCSQ, and IUS-12 according to parity. The scores of the two groups were presented with means (M) and the range of scores. Mean differences of them were assessed using Pearson *t*-test. Then, linear regression analysis was employed to evaluate the predictors of FOC in nulliparous and multiparous women, respectively. The level of statistical significance was set at *p* < 0.05.

## Results

### Participant Characteristics

There were 969 valid questionnaires, including 615 (63.5%) nulliparas and 354 (36.5%) multiparas. The mean age of pregnant women was 30.1 (SD = 3.8). Most of the participants were pregnant at 12–28 weeks (*n* = 620), full-time workers (*n* = 638), middle-income households of China (*n* = 378), and with college or above educational background (*n* = 794). Of the 969 pregnant women, 67.1% (*n* = 650) were planned pregnancy, 27.5% (*n* = 266) were unplanned pregnancy, and 5.5% (*n* = 53) were assisted reproductive technology pregnancy. Most of the participants received full support from their spouses (87.2%), while 11.1% were general support and 1.7% had little support.

Furthermore, chi-square tests showed that there were significant differences between the fear of childbirth group (CAQ ≥ 28) and no fear of childbirth group (CAQ < 28) on the part of maternal age, parity, planning of pregnancy, and prenatal spousal support (all *P*-values < 0.05). Additional sociodemographic and obstetric variables are shown in Table 1.

**Table 1:** Sociodemographic characteristics of participants (*N* = 969).

	***n* (%)**	**CAQ < 28** ***n* (%)**	**CAQ ≥ 28** ***n* (%)**	***χ^2^***	***P***
**Age**				9.951	0.019
20–25	114	35	79		
26–30	402	109	293		
31–35	385	144	241		
36–40	68	24	44		
**Employment status**				5.606	0.231
Full-time	638	201	437		
Part-time	42	11	31		
Unemployed	261	92	169		
Peasant	21	8	13		
Student	7	0	7		
**Educational level**				1.990	0.575
Junior school or below	42	17	25		
Senior school	133	45	88		
College	694	221	473		
Postgraduate or above	100	29	71		
**Monthly income (CNY)**				3.268	0.352
<5,000	350	101	249		
5,000–7,999	378	125	253		
8,000–9,999	110	39	71		
>10,000	131	47	84		
**Marital status**				1.260	0.262
Married	951	304	647		
Single	18	8	10		
**Residence**				0.346	0.557
Urban	802	255	547		
Rural	167	57	110		
**Medical insurance**				0.554	0.457
Yes	846	276	570		
No	123	36	87		
**Gestational week**				0.233	0.629
12–28	620	203	417		
29–40	349	109	240		
**Parity**					
Nullipara	615	164	451	23.594	0.000
Multipara	354	148	206		
**Planning of pregnancy**				24.293	0.000
Planned	650	233	417		
Unplanned	266	55	211		
ART	53	24	29		
**Family's opinion of childbirth mode**				3.184	0.204
Spontaneous childbirth	599	193	406		
CS	105	41	64		
Neutral	265				
**Preferred mode of childbirth**				4.577	0.101
Spontaneous childbirth	610	198	412		
CS	129	50	79		
Neutral	230	64	166		
**Prenatal spousal support**				11.012	0.004
Very little support	16	4	12		
General support	108	20	88		
Full support	845	288	557		
**Access to childbirth information**				5.710	0.127
Hospital	208	74	134		
Book/Newspaper/Magazine	33	10	23		
Internet/Applications of smartphone	544	159	385		
Friends/Families	184	69	115		
**Regular prenatal visit**				0.639	0.424
Yes	952	305	647		
No	17	7	10		
**Mode of previous birth** ^**a**^				4.444	0.217
Spontaneous childbirth	175	70	105		
Instrumental vaginal birth	9	2	7		
Elective CS	90	45	45		
Emergency CS	80	31	49		
**Epidural analgesia during previous labor^a^**				0.235	0.889
No	194	79	115		
Yes	149	64	85		
Other	11	5	6		

*CAQ, childbirth attitude questionnaire; CNY, Chinese Yuan; ART, assisted reproductive technology; CS, cesarean section*.

a *Multiparous woman: N = 314*.

### Descriptive Statistics of Measurements

#### Total

The results of the scores of the CAQ, SCSQ, and IUS-12 among 969 pregnant women are shown in Table 2. The mean score of the CAQ was 32.76 (SD = 9.842), and 67.8% (*n* = 657) of women had FOC symptoms with a CAQ score ≥ 28. The percentages of women with mild (score of 28–39), moderate (40–51), and severe FOC (52–64) were 43.6, 20.2, and 4.0%, respectively. The mean scores of the SCSQ-P and SCSQ-N were 21.32 (SD = 7.025) and 9.12 (SD = 4.740), respectively. The mean IUS-12 score was 23.81 (SD = 6.099), and 52.1% of women (*n* = 505) scored higher than average.

**Table 2:** Pearson correlation analysis and descriptive statistics of main variables.

	**Range**	**Mean (SD)**	**1**	**2**	**3**	**4**	**5**	**6**	**7**	**8**	**9**	**10**	**11**
1. CAQ (total score)	16–64	32.76 (9.842)											
2. CAQ-FBS	5–20	11.46 (3.649)	0.917^**^										
3. CAQ-FLP	4–16	8.24 (2.875)	0.915^**^	0.761^**^									
4. CAQ-FLC	4–16	8.37 (2.698)	0.907^**^	0.771^**^	0.783^**^								
5. CAQ-FE	3–12	4.69 (1.783)	0.795^**^	0.623^**^	0.696^**^	0.656^**^							
6. SCSQ-P	1–36	21.32 (7.025)	−0.071^*^	0.000	−0.094^**^	−0.063^*^	−0.144^**^						
7. SCSQ-N	0–24	9.12 (4.740)	0.375^**^	0.341^**^	0.339^**^	0.332^**^	0.325^**^	0.179^**^					
8. IUS (total score)	12–56	23.81 (6.099)	0.397^**^	0.373^**^	0.348^**^	0.332^**^	0.367^**^	0.020	0.404^**^				
9. IUS-PA	6–28	10.58 (3.622)	0.389^**^	0.338^**^	0.349^**^	0.324^**^	0.405^**^	−0.103^**^	0.378^**^	0.899^**^			
10. IUS-IA	3–15	7.12 (2.144)	0.136^**^	0.166^**^	0.113^**^	0.107^**^	0.069^*^	0.194^**^	0.189^**^	0.597^**^	0.258^**^		
11. IUS-PE	3–15	6.11 (1.890)	0.382^**^	0.368^**^	0.327^**^	0.328^**^	0.330^**^	0.042	0.364^**^	0.826^**^	0.692^**^	0.300^**^	

*CAQ, childbirth attitude questionnaire, four dimensions of CAQ; FBS, fear of baby safety, FLP, fear of labor pain, FLC, fear of losing control, FE, fear of the environment; SCSQ, simplified coping style questionnaire, two dimensions of SCSQ; SCSQ-P, positive coping, SCSQ-N, negative coping; IUS-12, intolerance of uncertainty scale-12, three dimensions of IUS-12; IUS-PA, prospective action, IUS-IA, inhibitory action, IUS-PE, prospective emotion; SD, standard deviation*.

* *p < 0.05, 2-tailed*.

** *p < 0.01, 2-tailed*.

#### Mean Differences Across CAQ, SCSQ, and IUS-12 in Parity

The mean differences across CAQ, SCSQ, and IUS-12 between nulliparous and multiparous women are shown in Table 3. It was investigated that nulliparous and multiparous women differed significantly in the sum scores of the CAQ, the SCSQ-P, and SCSQ-N (all *P*-values < 0.05). There were no significant differences in the sum scores of IUS-12 between nulliparous and multiparous women.

**Table 3:** Mean differences between nulliparous and multiparous women across CAQ, SCSQ, and IUS-12.

	**Nulliparous (*N* = 615)**		**Multiparous (*N* = 314)**			
	**Mean (SD)**	**Range**	**Mean (SD)**	**Range**	***t***	***P***
CAQ (total score)	34.05 (9.871)	16–64	30.51 (9.391)	16–60	5.534	0.000
CAQ-FBS	11.88 (3.611)	5–20	10.72 (3.600)	5–20	4.847	0.000
CAQ-FLP	8.57 (2.924)	4–16	7.68 (2.699)	4–15	4.808	0.000
CAQ-FLC	8.82 (2.648)	4–16	7.58 (2.602)	4–15	7.127	0.000
CAQ-FE	4.77 (1.834)	3–12	4.54 (1.685)	3–11	1.977	0.048
SCSQ-P	21.71 (6.904)	1–36	20.65 (7.190)	1–36	2.246	0.025
SCSQ-N	9.44 (4.865)	0–24	8.58 (4.468)	0–23	2.790	0.005
IUS-12 (total score)	23.77 (6.155)	12–54	23.88 (6.008)	12–56	−0.271	0.787
IUS-PA	10.49 (3.675)	6–27	10.73 (3.530)	6–28	−1.013	0.311
IUS-IA	7.11 (2.203)	3–15	7.15 (2.042)	3–15	−0.282	0.778
IUS-PE	6.17 (1.874)	3–15	6.00 (1.915)	3–15	1.360	0.174

*CAQ, childbirth attitude questionnaire, four dimensions of CAQ; FBS, fear of baby safety, FLP, fear of labor pain; FLC, fear of losing control; FE, fear of the environment; SCSQ, simplified coping style questionnaire, two dimensions of SCSQ; SCSQ-P, positive coping; SCSQ-N, negative coping; IUS-12, intolerance of uncertainty scale-12, three dimensions of IUS-12; IUS-PA, prospective action; IUS-IA, inhibitory action; IUS-PE, prospective emotion; SD, standard deviation*.

### Correlations Analyses

The results of correlational analyses are shown in Table 2. The sum scores of CAQ positively correlated with negative coping styles (*r* = 0.375, *p* < 0.01) and IU (*r* = 0.397, *p* < 0.01) while negatively related with positive coping styles (*r* = −0.071, *p* < 0.05). Furthermore, the sum scores of SCSQ-N correlated with IUS-12 significantly (*r* = 0.404, *p* < 0.01). However, there was no significant correlation between the sum scores of SCSQ-P and IUS-12.

### Hierarchical Linear Regression for Predicting FOC

The results are shown in Table 4. It was investigated that the sociodemographic and obstetric characteristics could explain 8.5% of the variation in FOC (Model 1: *F* = 5.532, *R*^2^ = 0.085, *p* = 0.000). Model 2 (*F* = 5.708, *R*^2^ = 0.093, *p* = 0.005) could explain 9.3% of the variation in FOC, of which 0.8% were explained by the sum score of SCSQ-P. In Model 3 (*F* = 15.119, *R*^2^ = 0.223, *p* = 0.000), all variables explained 22.3% of the variation in FOC. Additionally, SCSQ-P (β = −0.153, *p* < 0.000) and SCSQ- N (β = 0.375, *p* < 0.000) were significant risk factors of the FOC. In Model 4 (*F* = 19.808, *R*^2^ = 0.284, *p* = 0.000), all the included variables explained 28.4% of the variation in FOC, of which 6.1% were explained by the sum score of IUS-12. In addition, the influences of the score of SCSQ-P (β = −0.135, *p* < 0.000), SCSQ-N (β = 0.261, *p* < 0.000), and IUS-12 (β = 0.277, *p* < 0.000) were significant.

**Table 4:** Results of hierarchical linear regression analysis for predicting FOC (*N* = 969).

**Variables**	**Model 1**			**Model 2**			**Model 3**			**Model 4**		
	***B***	**β**	***P***	***B***	**β**	***P***	***B***	**β**	***P***	***B***	**β**	***P***
**Age (Ref: 20–25)**												
26–30	1.945	0.097	0.061	2.238	0.112	0.031	2.327	0.117	0.016	1.341	0.067	0.149
31–35	0.703	0.035	0.523	1.109	0.055	0.317	1.170	0.058	0.254	0.364	0.018	0.713
36–40	2.254	0.059	0.148	2.512	0.065	0.106	2.001	0.052	0.165	1.298	0.034	0.349
**Gestational weeks (Ref: 12–28)**												
29–40	−1.385	−0.048	0.416	−1.215	−0.043	0.474	−1.130	−0.040	0.472	−0.602	−0.021	0.690
**Educational level (Ref: Junior school or below)**												
Senior school	−0.194	−0.009	0.901	0.300	0.014	0.848	−0.429	−0.020	0.767	−0.185	−0.008	0.894
College	0.411	0.013	0.821	1.165	0.036	0.523	0.779	0.024	0.645	0.638	0.020	0.694
Postgraduate or above	−0.265	−0.013	0.679	−0.286	−0.014	0.654	0.070	0.003	0.906	0.034	0.002	0.953
**Parity (Ref: Nulliparous)**												
Multiparous	−4.162	−0.204	0.000	−4.251	−0.208	0.000	−3.693	−0.181	0.000	−3.806	−0.186	0.000
**Planning of pregnancy (Ref: Planned)**												
Unplanned	2.803	0.127	0.000	2.750	0.125	0.000	2.430	0.110	0.000	2.109	0.096	0.001
ART	−2.005	−0.046	0.154	−2.105	−0.049	0.134	−2.819	−0.065	0.030	−2.410	−0.056	0.054
**Family's opinion of childbirth mode (Ref: Spontaneous childbirth)**												
CS	1.394	0.044	0.416	1.078	0.034	0.529	2.159	0.068	0.174	0.689	0.022	0.654
Neutral	0.963	0.044	0.285	0.844	0.038	0.347	1.239	0.056	0.137	0.774	0.035	0.333
**Preferred mode of childbirth (Ref: Spontaneous childbirth)**												
CS	0.713	0.025	0.652	0.933	0.032	0.554	−0.248	−0.009	0.865	0.787	0.027	0.577
Neutral	1.675	0.072	0.068	1.883	0.081	0.041	0.919	0.040	0.282	0.949	0.041	0.247
**Prenatal spousal support (Ref: Very little support)**												
General support	−2.416	−0.077	0.347	−2.262	−0.072	0.377	−3.082	−0.099	0.194	−4.336	−0.139	0.058
Full support	−5.490	−0.186	0.024	−5.173	−0.176	0.033	−4.766	−0.162	0.034	−5.577	−0.189	0.010
SCSQ-P^a^				−0.127	−0.091	0.005	−0.215	−0.153	0.000	−0.188	−0.135	0.000
SCSQ-N^a^							0.779	0.375	0.000	0.541	0.261	0.000
IUS-12^a^										0.446	0.277	0.000
*R^2^*		0.085			0.093			0.223			0.284	
*ΔR^2^*		0.070			0.076			0.208			0.270	
*F*		5.532			5.708			15.119			19.808	
*P*		0.000			0.005			0.000			0.000	

*Ref, reference; ART, assisted reproductive technology; CS, cesarean section; two dimensions of SCSQ: SCSQ-P, positive coping, SCSQ-N, negative coping; IUS-12, intolerance of uncertainty scale-12; B: unstandardized coefficients; β: standardized coefficients*.

a *Continuous variable*.

Table 5 shows the results of linear regression for predicting FOC among nulliparous women (*F* = 46.081, adjusted *R*^2^ = 0.269). It was worth noting that higher scores of CAQ were significantly associated with unplanned pregnancy (β = 0.106, *p* = 0.003), a lower score of SCSQ-P (β = −0.108, *p* = 0.002), a higher score of SCSQ-N (β = 0.298, *p* = 0.000) and IUS-12 (β = 0.315, *p* = 0.000).

**Table 5:** Results of linear regression for predicting FOC of nulliparous women (*N* = 354).

	**Unstandardized coefficients**		**Standardized coefficients**		**95% CI for *B***			
	***B***	**Std. error**	**β**	***P***	**Lower**	**Upper**	***F***	***ΔR^2^***
**Employment status (Ref: Full-time)**							8.636^***^	0.219^***^
Part-time	6.037	2.075	0.141	0.004	1.955	10.119		
Unemployed	−1.335	1.033	−0.064	0.197	−3.368	0.697		
Peasant	0.688	2.050	0.017	0.738	−3.345	4.720		
**Planning of pregnancy (Ref: Planned)**								
Unplanned	2.323	0.943	0.119	0.014	0.469	4.178		
ART	−3.617	2.498	−0.070	0.148	−8.531	1.296		
**Mode of previous birth (Ref: Spontaneous childbirth)**								
Instrumental vaginal birth	0.357	2.871	0.006	0.901	−5.291	6.005		
Elective CS	−1.945	1.114	−0.09	0.082	−4.136	0.246		
Emergency CS	0.550	1.147	0.025	0.632	−1.705	2.806		
**Epidural analgesia during previous labor (Ref: No)**								
Yes	−0.804	0.920	−0.042	0.383	−2.614	1.007		
Other	−4.977	2.635	−0.092	0.060	−10.161	0.207		
SCSQ-P^a^	−0.244	0.066	−0.187	0.000	−0.374	−0.113		
SCSQ-N^a^	0.404	0.118	0.192	0.001	0.171	0.637		
IUS-12^a^	0.427	0.086	0.273	0.000	0.259	0.596		

*Ref, reference; ART, assisted reproductive technology; CS, cesarean section; two dimensions of SCSQ: SCSQ-P, positive coping, SCSQ-N, negative coping; IUS-12, intolerance of uncertainty scale-12; B, unstandardized coefficients; β, standardized coefficients*.

a *Continuous variable*.

*** *p < 0.001, 2-tailed*.

Table 6 reveals that part-time workers (β = 0.141, *p* = 0.004), unplanned pregnancy (β = 0.119, *p* = 0.014), the score of SCSQ-P (β = −0.187, *p* = 0.000), SCSQ-N (β = 0.192, *p* = 0.001), and IUS-12 (β = 0.273, *p* = 0.000) were risk factors of FOC among multiparous women.

**Table 6:** Results of linear regression for predicting FOC of multiparous women (*N* = 615).

	**Unstandardized coefficients**		**Standardized coefficients**		**95% CI for** ***B***		***F***	***ΔR^2^***
	***B***	**Std. Error**	**β**	***P***	**Lower**	**Upper**		
Planning of pregnancy (Ref: Planned)							46.081^***^	0.269^***^
Unplanned	2.526	0.833	0.106	0.003	0.890	4.161		
ART	−1.481	1.388	−0.037	0.286	−4.206	1.244		
SCSQ-P^a^	−0.155	0.050	−0.108	0.002	−0.253	−0.057		
SCSQ-N^a^	0.604	0.077	0.298	0.000	0.454	0.754		
IUS-12^a^	0.505	0.060	0.315	0.000	0.387	0.622		

*Ref, reference; ART, assisted reproductive technology; two dimensions of SCSQ: SCSQ-P, positive coping, SCSQ-N, negative coping; IUS-12, intolerance of uncertainty scale-12; B, unstandardized coefficients; β, standardized coefficients*.

a *Continuous variable*.

*** *p < 0.001, 2-tailed*.

## Discussion

Fear of childbirth has become one of the most common psychological symptoms. It is confirmed that FOC leads to significant anxiety, depression, and loneliness (1, 2, 4, 52). We found that the prevalence of FOC was 67.8% (Childbirth Attitude Questionnaire, CAQ ≥ 28) in the present study, which is consistent with one research in Chongqing, China (67.1%) (32) and a survey in Guangdong, China (79.4%) (53). However, there were lower incidences of FOC reported in Sweden (24.6%) and Italy (8.2%) before the outbreak of the COVID-19 pandemic (13, 54). Moreover, approximately 24.2% of the sample reported high or severe FOC in this study. In addition, IU and negative coping styles were positively correlated with the sum score of CAQ. However, it is investigated that the positive coping style of pregnant women was inversely related to fear of childbirth. According to the results, it may be helpful for pregnant women with FOC symptoms to adopt positive coping styles and decrease the level of intolerance of uncertainty.

Since the COVID-19 pandemic was declared by the World Health Organization (WHO) (55), a growing body of evidence has shown that pregnant women have experienced more psychological symptoms (14, 16, 21). Because of the heterogeneity of measurement tools of FOC, several studies reported lower rates of moderate FOC among pregnant women during the pandemic: 10.% in Portugal and 31.% in Iran (2, 56). Therefore, a multicenter cross-sectional survey and a larger sample are necessary to explore the incidence and predictors of FOC during the COVID-19 pandemic. Although the potential risk factors of FOC are widely demonstrated and reported in previous studies from various countries, there is a lack of research validating the relationships between intolerance of uncertainty, coping styles, and FOC in China.

Our study identified that primiparas, unplanned pregnancy, and little spousal support were significant risk factors in FOC. In line with previous research (2, 44, 57), primiparity was one of the significant predictors of severe FOC. In contrast, one Finland population-based analysis suggested that multiparous women also tended to have fear of childbirth for previous cesarean section, preterm birth, and unspecified socioeconomic status (58). Given that parity was the strong predictor of FOC, we compared different risk factors of FOC between nulliparous and multiparous. According to the results, unplanned pregnancy, negative coping style, and IU were predictors of FOC in both nulliparous and multiparous. Of note, unplanned pregnant women tend to score higher on FOC (59, 60). They may consider this pregnancy unintended or unwanted, which is associated with a sense of being unprepared to be a mother (44, 59). At the same time, part-time work was significantly related to FOC in multiparous. The uncertain income and poor financial status of pregnant women may contribute to life stress. Similar to the present study, a lack of spousal support relates to an increased probability of FOC (44, 61). However, Bilgin et al. argued that there is no significant relationship between spousal support and FOC (62). Because the samples and measurement tools are different, various studies have inconsistent results. Therefore, further research is warranted in different countries and regions to specify these associations in the present study.

According to the results, 26.5% of women adopt negative coping styles (Simplified Coping Style Questionnaire-Negative coping styles, SCSQ-N ≥ 12). A similar result was obtained in a cross-sectional survey during the pandemic (16). In addition, it is confirmed that passive coping strategies of pregnant women are correlated with depression, anxiety, and psychological distress (63). Daglar et al. also suggested that optimistic coping styles with stress were correlated with pregnant women's self-confidence (64). Interestingly, our study investigated that negative coping styles were risk factors in FOC, while positive coping styles were protective factors. Therefore, positive coping styles may be associated with better mental resiliency and helpful for women to face distress during pregnancy.

In addition, the present study confirmed that IU was positively related to FOC, consistent with the survey conducted by Rondung et al. (13). The uncertainty caused by the pandemic may increase pregnant women's fear of childbirth and worry about being infected with the virus. The unpredictability caused by pandemic restrictions may increase pregnant women's fears and worries about childbirth. Pregnant women with high IU would experience significant distress and anxiety and take different coping strategies when faced with ambiguous circumstances (22, 24, 65).

A recent review has suggested that non-pharmacological treatments may reduce the fear of childbirth and cesarean section births (66). In light of the evidence about alleviating the FOC of pregnant women, psychoeducation intervention based on Human Caring Theory, online cognitive behavior therapy, mindfulness training, art therapy, and haptotherapy effectively decrease the level of FOC (3, 30, 67–70). Furthermore, access to childbirth information was associated with a decreased likelihood of fear of birth and cesarean delivery (71). It is investigated that sufficient information about childbirth and positive coping strategies (e.g., mindfulness training, psychoeducation training, art therapy) may decrease the IU and FOC of pregnant women.

To some extent, the present study further highlights the impact and risk factors of FOC among pregnant women. It may provide a new perspective to construct the targeted interventions of FOC. The current findings have important implications for the assessment and treatment of fear of childbirth among pregnant women. It is indicated that primiparas, unplanned pregnancy, few spousal support, intolerance of uncertainty, and negative coping styles were significant risk factors in FOC. Women who adopt positive coping strategies experience a lower level of childbirth fear. It is suggested that more attention should be paid to identifying pregnant women with the high level of IU, especially for nulliparous. For instance, knowing that pregnant women with high IU tend to adopt negative coping styles, clinicians could make more empirically informed decisions to intervene in psychological treatment.

However, as a cross-sectional study, the universality of the results was limited, and the long-term effects of COVID-19 on pregnant women may not be inferred using the current results. Thus, there are several limitations of our study that should be noted. First, the convenient sampling method may lead to certain methodological limitations. The small number of participants in some subgroups may induce sample selection bias. Future studies need a larger sample to establish and confirm causal relationships among FOC, IU, and coping styles. Second, the data were self-report without external observation. Therefore, FOC, IU, and coping styles of pregnant women cannot be considered the diagnosis of psychopathology. In addition, the cut-off scores of measurements in this study should be investigated in the future. Third, although most of the differences in background variables were controlled, some variables (e.g., personality, pregnancy status) cannot be controlled. Fourth, because the level of fear of COVID-19 in pregnant women was not measured in this study, it is difficult to infer how the pandemic affected the main variables in the study. Furthermore, the survey did not include other negative emotionality variables (e.g., trait anxiety, neuroticism, depression). Thus, these results may not be specific to IU and negative coping styles. As such, our results should be replicated in a larger sample to verify the relations between IU, the coping styles, and FOC of pregnant women. Therefore, future research should pay more attention to prenatal psychological symptoms screening and the construction of targeted interventions for FOC.

## Conclusion

In summary, the results revealed that the prevalence of FOC was 67.8% among 969 Chinese pregnant women. It is identified that primiparas, unplanned pregnancy, few spousal support, intolerance of uncertainty, and negative coping styles were significant risk factors in FOC. Thus, more attention should be paid to screening perinatal psychological symptoms, such as the high level of intolerance of uncertainty and negative coping styles. Future research should be conducted to verify our findings within a large and cross-regional sample, and a longitudinal study is necessary.

## Data Availability Statement

The raw data supporting the conclusions of this article will be made available by the authors, without undue reservation.

## Ethics Statement

The research was conducted in accordance with the Declaration of Helsinki. The study was supported by the Ethics Committee of the Third Affiliated Hospital of Zhengzhou University and the Chinese Health Department (No. 2021-169-01). Written consent was obtained from the participants, and they voluntarily participated in this study.

## Author Contributions

LH contributed to the design of the study, acquisition of data, and revision of the manuscript. HB critically reviewed the manuscript and provided English edits. BL contributed to the acquisition of data and revision of the manuscript. YL and YW contributed to the acquisition and interpretation of data. All the authors read and approved the final manuscript.

## Funding

This research was supported by the Joint Construction Project of Henan Province's Medical Science and Technology Research Plan (No. SBGJ2018049).

## Conflict of Interest

The authors declare that the research was conducted in the absence of any commercial or financial relationships that could be construed as a potential conflict of interest.

## Publisher's Note

All claims expressed in this article are solely those of the authors and do not necessarily represent those of their affiliated organizations, or those of the publisher, the editors and the reviewers. Any product that may be evaluated in this article, or claim that may be made by its manufacturer, is not guaranteed or endorsed by the publisher.
